# Therapists’ Perceptions of Social Media and Video Game Technologies in Upper Limb Rehabilitation

**DOI:** 10.2196/games.3401

**Published:** 2015-03-10

**Authors:** Sandy K Tatla, Navid Shirzad, Keith R Lohse, Naznin Virji-Babul, Alison M Hoens, Liisa Holsti, Linda C Li, Kimberly J Miller, Melanie Y Lam, HF Machiel Van der Loos

**Affiliations:** ^1^Sunny Hill Health Centre for ChildrenVancouver, BCCanada; ^2^The Department of Occupational Science and Occupational TherapyUniversity of British ColumbiaVancouver, BCCanada; ^3^Biomedical Engineering Graduate ProgramUniversity of British ColumbiaVancouver, BCCanada; ^4^School of KinesiologyAuburn UniversityAuburn, ALUnited States; ^5^Department of Physical TherapyUniversity of British ColumbiaVancouver, BCCanada; ^6^Child and Family Research InstituteVancouver, BCCanada; ^7^Arthritis Research Centre of CanadaVancouver, BCCanada; ^8^Department of Human KineticsSt Francis Xavier UniversityAntigonish, NSCanada; ^9^Department of Mechanical EngineeringUniversity of British ColumbiaVancouver, BCCanada

**Keywords:** virtual reality, technology adoption, rehabilitation, therapy, social media, gaming, stroke, cerebral palsy, hemiplegia

## Abstract

**Background:**

The application of technologies, such as video gaming and social media for rehabilitation, is garnering interest in the medical field. However, little research has examined clinicians’ perspectives regarding technology adoption by their clients.

**Objective:**

The objective of our study was to explore therapists’ perceptions of how young people and adults with hemiplegia use gaming and social media technologies in daily life and in rehabilitation, and to identify barriers to using these technologies in rehabilitation.

**Methods:**

We conducted two focus groups comprised of ten occupational therapists/physiotherapists who provide neurorehabilitation to individuals with hemiplegia secondary to stroke or cerebral palsy. Data was analyzed using inductive thematic analysis. The diffusion of innovations theory provided a framework to interpret emerging themes.

**Results:**

Therapists were using technology in a limited capacity. They identified barriers to using social media and gaming technology with their clients, including a lack of age appropriateness, privacy issues with social media, limited transfer of training, and a lack of accessibility of current systems. Therapists also questioned their role in the context of technology-based interventions. The opportunity for social interaction was perceived as a major benefit of integrated gaming and social media.

**Conclusions:**

This study reveals the complexities associated with adopting new technologies in clinical practice, including the need to consider both client and clinician factors. Despite reporting several challenges with applying gaming and social media technology with clinical populations, therapists identified opportunities for increased social interactions and were willing to help shape the development of an upper limb training system that could more readily meet the needs of clients with hemiplegia. By considering the needs of both therapists and clients, technology developers may increase the likelihood that clinicians will adopt innovative technologies.

## Introduction

Video games are a form of virtual reality systems that offer real-time, immersive, computer-based environments with which users interact and explore [1,2]. Video game systems have gained attention as components of rehabilitation. Studies have examined video gaming as an intervention to target a range of impairments, including balance, mobility, cognition, and upper extremity functioning [3-5]. Video games are a promising tool because they can provide challenging, repetitive, task-specific, reward based, and intensive conditions needed to promote brain remodelling after neurological injury [3]. Cerebral palsy (CP) and stroke are the leading causes of neurological disability in children [6] and in adults [7], respectively, and video game therapy is an emerging area of research with both these populations [8]. While knowledge regarding the effectiveness of gaming in rehabilitation is limited, findings to date are promising (for a recent review, see [9]). Gaming-based rehabilitation is safe and feasible [1], and controlled trials and cohort studies have demonstrated positive effects of gaming for upper extremity function both in adults post stroke and in children with CP [4,8,10].

Social media, a companion interactive technology, has transformed communication and is progressively influencing healthcare. Nevertheless, in contrast to video game, little research is available on the application of social media in the field of rehabilitation [11]. Social media refers to a wide variety of online platforms (eg, Facebook and Twitter) that allow users to exchange information, links, and opinions at a highly interactive and rapid pace [12]. In rehabilitation, social media is a platform with the potential to promote physical, cognitive, and psychosocial health outcomes. In recent years, the global adoption of social networking platforms by users has prompted scholars to explore how and why these sites are being used. These platforms are well suited for individuals with disabilities because they can reduce social isolation, which is identified as one of the most disabling limitations for this population [13,14].

While gaming technologies and social media offer much promise for improving clinical outcomes, limited research has explored perceptions of these technologies from the viewpoint of clinicians working in rehabilitation [4,15]. Numerous factors may influence a clinician’s decision to adopt these types of interventions in clinical practice. An analysis of gaming in rehabilitation revealed a number of barriers to adoption, including concerns about how to design effective, efficient and easy-to-learn systems, challenges with platform compatibility, immature engineering processes, ethical challenges, limited awareness and unrealistic expectations by clinicians, and perceptions that video game use eliminates the need for clinicians [16]. One study examined clinicians’ perspectives of adopting a rehabilitation specific system, GestureTek, and identified time-to-learn, knowledge of clinical applicability of the system, and poor patient motivation as barriers to its use, while educational opportunities and social influences were facilitators [17].

To date, scarce research has examined clinicians’ views and acceptance of commercially available or rehabilitation-specific video gaming technology as interventions [17,18]. Moreover, clinicians’ perspectives regarding social media are notably limited. Research is needed to understand the synergy between social media and evidence-based practice [19]. Because clinicians are instrumental in mediating clients’ use of technology for rehabilitation, it is pertinent to understand their perceptions. If their views are not understood or incorporated in the development of these technology-based interventions, use may be limited. Thus, the two purposes of this qualitative study were (1) to explore occupational and physical therapists’ perceptions of how young people and adults with hemiplegia use social media and gaming technology in daily life and rehabilitation, and (2) to identify barriers to the use of these technologies in rehabilitation. We applied the diffusion of innovations theory as a framework to explain factors influencing occupational and physical therapists’ adoption of these technologies with their clients. This theory provides a relevant framework for interpreting our research because it emphasizes users’ perceptions of the innovation, rather than the attributes of the innovation as most influential in understanding adoption decisions [20]. This theory proposes an innovation-adoption process that individuals, acting as decision making units, undergo, beginning with acquiring knowledge of an innovation, forming an attitude toward the innovation, then implementing the new idea, and finally seeking confirmation of the decision [21].

## Methods

### Study Setting

This study took place at two rehabilitation centres in British Columbia (BC), Canada. Hospital-based rehabilitation in BC is publicly funded, and residents have the option of obtaining additional private therapies under a fee-for-service provision model.

### Participants

Purposive sampling was used to recruit occupational therapists or physiotherapists with at least 1 year of experience who were currently providing rehabilitation to children with hemiplegia secondary to a diagnosis of CP or adults with hemiplegia secondary to stroke. A purposeful selection approach was used to achieve a heterogeneous sample of therapists who represented a broad spectrum of experiences and contexts in providing therapy to individuals with hemiplegia. Participants were recruited through managers and supervisors in different clinical settings (both public and private) via an email providing a brief description of the study. All participants provided informed consent. Ethical approval for this study was obtained from the local university ethical review boards (REB #: H12-00220).

### Data Collection

The 2 focus groups that were conducted at separate therapy facilities within the Lower Mainland of Vancouver, BC comprised (1) participants representing two publically funded child development and rehabilitation centres, and (2) a private clinic that provides outpatient therapy to both adults and children with neurological conditions.

Each focus group of between 4-6 participants lasted 90 minutes. Participants completed a questionnaire to provide demographic and clinical practice information. The focus groups were facilitated by the author Sandy K Tatla (ST), an occupational therapist with group facilitation and neurorehabilitation experience. The facilitator’s assumptions entering the focus group sessions were shaped by her experiences providing rehabilitation to clients and her interest in understanding factors that influence technology use in clinical practice. A research assistant generated field notes to offer an additional perspective of the focus group findings and to provide a nuanced context of each group, thereby providing insight about the behaviour of participants and their relationships and interactions with each other. During the introduction, the facilitator reiterated the purpose of the study and encouraged an open climate for all participants to express their opinions freely.

The discussion was based on a semi-structured focus group guide, which included a selection of open-ended questions developed in conjunction with a team of experts with experience using focus group methodology. The interview questions were pilot tested with five physical and occupational therapists and refined to ensure clarity (Textbox 1). The focus groups were audio-recorded and transcribed verbatim. The primary author (ST) reviewed all transcriptions to confirm the content and to identify any discrepancies in interpretation.

### Data Analysis

An inductive process of thematic analysis was used to analyze the focus group transcripts. This process offered a flexible and useful research tool to provide a rich and detailed account of the data and also permitted unanticipated insights to be generated [22].

Though flexibility is a key advantage of thematic analysis, a structured coding approach promotes consistency amongst individuals conducting the data analysis [22,23]. First, each individual familiarized themselves with the data by reading and re-reading each transcript and recording initial ideas (ST, NVB, KL, KM). Next, initial codes were generated by each individual to organize the data in a systematic way across the entire data set. Following initial coding, emerging themes were identified and data relevant to each potential theme were collated. Themes were then reviewed in pairs (ST and NVB; KL and KM) to determine if the themes related to the coded extracts and the entire data set, and the thematic map of the analysis were produced with consensus. Both pairs of "coders" met as a group, led by a researcher with expertise in qualitative research (LH), to refine the specifics of each theme. Finally, the primary author and focus group facilitator (ST) met with each participant to verify the accuracy and completeness of the themes. One participant was unavailable for member checking because of scheduling conflicts. The remaining nine attendees reported that the themes accurately represented the depth of discussion and cross section of opinions.

**Focus group questions**

Questions

- Please tell us about the typical upper extremity home exercises that you prescribe for patients.
  - Probes:
    - Desired range of motion, repetitions, frequency, duration.
    - Is equipment required?
    - Do your clients usually need someone to assist them with the exercise?

- Please tell us about the types of splints and other devices that your clients use to enable them to complete their upper extremity home exercises.
  - Probes:
    - Is equipment required?
    - Do your clients need someone to assist them with their exercises?
    - Weight, length, restrictions, for which activities, for what duration?

- To your knowledge, do your clients use social media, computerized programs or games at home?
  - If so, what types do they use?
    - Devices: iPads, desktops, iPhones, etc.
    - Systems: Wii, Kinect, etc.
    - Social media portals: Facebook, Twitter, Linked-in, Google Plus, Skype, YouTube,
    - Games: Super Mario, Wii fit, Angry Birds, Sudoku, Dance dance revolution, multiplayer games, Farmville etc.
  - How do they use them?
  - How frequently do they use them?
  - What kind of assistance would they require?
  - What are the barriers for your clients to using these tools?

- Tell us your thoughts about using social media to help motivate patients to practice their home exercise programs.
  - Probes:
    - Could it be beneficial? If so, why? If not, why not?
    - What might some of the obstacles / challenges be?
    - What features of social media would be important for the research team to incorporate in the design of a rehabilitation tool or the creation of a new tool?
    - What features of social media would be important for the research team to avoid in the design of a rehabilitation tool?
    - Anything else you wish to add?

- Tell us your thoughts about using games to help motivate patients to practice their home exercise programs.
  - Probes:
    - Could it be beneficial? If so, why? If not, why not?
    - What might some of the obstacles / challenges be?
    - What features of gaming would be important for the research team to incorporate in the design?
    - What features of gaming would be important for the research team to avoid in the design?
    - Anything else you wish to add?

- Tell us your thoughts about using robotics to help motivate patients to practice their home exercise programs.
  - Probes:
    - Could it be beneficial? If so, why?; If not, why not?
    - What might some of the obstacles / challenges be?
    - What features of robotics would be important for the research team to incorporate in the design?
    - What features of robotics would be important for the research team to avoid incorporating into the design?
    - Anything else you wish to add?

## Results

### Summary

Ten occupational and physical therapists drawn from three multidisciplinary rehabilitation centres participated in one of two focus groups. The sample contained 4 occupational therapists and 6 physiotherapists between the ages of 25-65, with a range of 3-25 years of experience providing therapy (Table 1). Participants in each focus group were employed in various contexts, including the school system, in-patient hospital settings, and community-based settings (either in a clinic or at clients’ homes). Based on Canadian statistics, our sample was representative of the clinician population [24].

**Table 1:** Demographic characteristics of participants (N=10).

Characteristics		n (%)
**Gender**		
	Males	2 (20%)
	Females	8 (80%)
**Age (years)**		
	20-34	5 (50%)
	35-49	4 (40%)
	50-64	1 (10%)
**Profession**		
	Occupational therapist	4 (40%)
	Physiotherapist	6 (60%)
**Primary practice area**		
	Neurorehabilitation (general)	5 (50%)
	Neurorehabilitation (pediatric)	4 (40%)
	Orthopedic rehabilitation	1 (10%)
**Experience (years)**		
	<5	4 (40%)
	6-9	1 (10%)
	10-14	2 (20%)
	15-19	1 (10%)
	20-24	0 (0%)
	>25	2 (20%)

Nine key themes associated with therapists’ perceptions of how clients use technology in daily life and rehabilitation were identified (Textbox 2). These nine themes were qualitatively clustered into the following three groups (1) use of social media and gaming technologies in daily life and rehabilitation of persons with hemiplegia, (2) the barriers to the application of the these technologies for rehabilitation, and (3) the potential benefits and the desirable features of video gaming and social media platforms for rehabilitation. Thematic analysis of the focus group findings demonstrated the presence of recurrent concepts and ideas, which was verified by all coders, indicating that data saturation was achieved with our sample.

**Identified themes and sub-themes**

Themes and sub-themes

- Use of social media and gaming technologies in daily life and rehabilitation of persons with hemiplegia
  - Limited use of video gaming and social media in therapy
  - Clients vary in their use of gaming and social media in their personal lives

- The barriers to implementing social media and video gaming for therapy
  - Age appropriateness of social media use
  - Privacy concerns related to social media use
  - Transfer of training
  - Lack of accessibility of gaming systems to meet clients’ needs
  - Reconciling therapist role within the gaming context

- Potential Benefits and Desirable Features
  - Social-Emotional Features
  - Rehabilitation Features
  - Usability Features

### Use of Social Media and Gaming Technologies in Daily Life and Rehabilitation of Persons With Hemiplegia

#### Limited Use of Video Gaming and Social Media in Therapy

Clinicians shared that they used video gaming to target lower but not upper limb training because their clients become frustrated with trying to use their impaired hand.

I almost never use gaming for the upper extremity. I almost exclusively use the Wii balance board, and very rarely. I get them to use just [their lower extremity], because usually if they are that affected, it’s quite frustrating to try to use the controller with the upper extremity.

P8, Group 2

Clinicians working with children reported that the integration of motivating activities was not specific to video gaming or other technology, but rather was an inherent part of how they deliver therapy. Therefore, video gaming was one of many different ways of motivating clients for therapy.

You can make up a game with zero equipment, you can use whatever families have in the house, too. So, really, tailoring it to what is of interest to the child and getting them involved.

P2, Group 1

However, the therapists also shared that tablets were used specifically for their motivating features during therapy.

the one thing that got the child to use their severely neglected arm to do anything was a little assist under his hand from his mom while he pushed the animals on the iPad, and I was like dancing and singing and had lots of fun toys, but the iPad was what got him to move his arm.

P4, Group 1

#### Clients Vary in Their Use of Gaming and Social Media in their Personal Lives

Overall, therapists said the types of video gaming and social media platforms that were used by their clients varied based on clients’ interests, values, and resources. For example, therapists perceived that some pediatric clients value and choose to purchase tablets. “Many of the parents have decided the iPad is really important and they’ve invested in it.” [P2, Group 1]. The user-friendly interface, interactive games, and educational applications on tablets were perceived as appropriate for children. Computers and tablets for personal gaming and communication were also important for some adult clients.

I’ve had a few younger stroke clients in their 40s and 50s, and the number one thing they mention to me is “I want to be able to use my computer. I want to play solitaire and I need to update my Facebook so my friends know what’s happening to me.

P6, Group 2

However, therapists found that some clients had no interest in using any of these forms of technology. "…the majority of my clients are adults and I’d say none of them use technology." [P9, Group 2]

### The Barriers to Implementing Social Media and Video Gaming for Therapy

#### Age Appropriateness of Social Media Use

The therapists revealed concerns about the age appropriateness of social media, a factor that prevented them from using video gaming on social media sites with their clients*.* “One of the things that really came to mind for me was the age and I think that we really need to be considering that kids under 13 are not allowed on Facebook.” [P4, Group 1] Another participant shared her perception that her adult clients prefer to use email and view social media sites as inappropriate for their age-group.

They’re sitting in front of their computer emailing everybody, but they’re not using Facebook. I know a lot of them consider it to be a child’s thing. Like a lot of them have this thing where, that’s what my kids do. That’s not what I do.

P7, Group 2

#### Privacy Concerns Related to Social Media Use

Therapists considered their role in protecting clients’ confidentiality and questioned using social media as part of rehabilitation because of concerns about privacy and security protection. "…if we are talking to the patient as a therapist… how much control do we have as therapists about who gets their information and how it is shared because there is always that issue of confidentiality." [P3, Group 2] For younger clients, therapists considered the challenges of resolving parents’ concerns with the children’s needs for autonomy.

If parents and therapists are deciding what games to choose for a particular child, how do you manage what’s available with what the children can access? Parents want to know how much control they can exert and if they will be able to ‘protect’ their child from what they don’t want them involved in…and yet the autonomy and access for those older kids is going to be a big piece of the motivation for them…

P4, Group 1

#### Transfer of Training

Therapists in this study also questioned how to achieve a balance between video gaming and other forms of therapy and how video gaming fits within traditional forms of therapy.

If, as a therapist, you are focused on the social media, and playing games, you’re going to start moving away from true participation in therapy. A movement game where clients are sitting in front of the screen isn’t the same as going outside and moving their bodies.

P2, Group 1

#### Lack of Accessibility of Gaming Systems to Meet Clients’ Needs

Therapists perceived a gap in the gaming market for individuals who have physical and cognitive limitations and saw challenges with implementing gaming in therapy related to cost, equipment requirements, and set-up. They also felt a lack of tailoring prevailed within the games and gaming interfaces that limited individualization to meet the varying physical, cognitive, social and developmental needs of clients with CP or stroke. "If they have to spend too much time learning the game, I think they’ll get frustrated and move on, and a lot of my clients won’t be able to do it." [P9, Group 2]. "The trickiness is to find the ‘just right’ challenge. Each individual is so different, has such different needs so that’s, I mean, a huge task." [P4, Group 1]. Some therapists were concerned about overuse injuries and compensatory movements that could create negative effects for clients. "We need to consider how clients can use compensatory strategies to play the games, and that can be harmful for their recovery." [P5, Group 2] Another shared, "One issue that I’ve run into specifically with some systems is that it doesn’t seem to detect the motion of somebody in a wheelchair." [P6, Group 2] Therapists in this study perceived commercial systems were not accessible in meeting the unique needs of their clients.

#### Reconciling Therapist Role Within the Gaming Context

Therapists also acknowledged that a lack of openness to changing how they delivered therapy could be an impediment to using gaming technologies. A therapist expressed the process she went through to adjust to a less active role when more sophisticated technology was integrated into therapy.

Therapeutically, ten minutes is a long time, like you kind of feel like you need to be doing something as a therapist that feels therapeutic. But for them to do it, I really did see the value of them again learning a strategy and then implementing the strategy and seeing the success of that strategy, which helped me as a therapist to say, okay, slow down, you don’t have to be in this front end pace, just let them be. Therapy is happening, even if you are not doing a lot, even though I am sitting and giving them verbal cues, therapy is still happening, and that was a bit of a shift for me as a therapist. We’re used to setting up cones, and you know, they might be doing something, but we’re moving the cones so we feel as therapists, yes, we are providing therapy, because I, my values are moving the cones… right?

P10, Group 2

This therapist’s experience describes psychological processes associated with behavioral change that therapists may undergo as they adjust to integrating technology and different approaches to their practice.

### Potential Benefits and Desirable Features

#### The Potential of Gaming and Social Media to Promote Opportunities for Social Connection and Increase Motivation for Practice

All therapists' perceived social connection with peers was a key benefit of clients using video gaming embedded in social media. They also felt that the ability of clients to engage in gaming and social media in their own homes would help clients feel safe to interact with their peers in an environment where their disability was not obvious to others.

Having your peers involved is huge… A lot of our kids don’t get to participate in normal activities because of their limitations. So this is normal and nobody has to be involved in seeing the adaptions that child may be trying to use, it’s just a normal activity.

P1, Group 1

Another said,

Because they can link up with other people, children, family members and in a way, compete in a non-competitive environment. So they can be involved in a game whether they are getting assistance or not, and the person doesn’t have to be anywhere near them and it’s not stressful because often for these kids it is stressful when they are in a group together. But here, they can be involved in Farmville, or something, when they couldn’t potentially be involved in their local basketball team.

P4, Group 1

Therapists considered the social networking opportunities for clients as a potentially strong motivator for increased practice and adherence to therapy.

I think the interesting thing about compliance too, people tend to do that if it’s more like a social thing, so you know, people will go to a gym if their friend will go with them. So if there was some way to kind of get social, I think they would be more motivated to do it.

P8, Group 2

Another said, "It would be interesting for those that are interested as a way to connect with other stroke survivors.

P6, Group 2

Therapists also shared some of their experiences with gaming being a potential motivator for more frequent practice.

I think it works. I think, honestly, I have a kid. I can’t get him to stand for like a minute and then I take him in front of a Wii and he will stand for forty-five. You see, it works. It’s super humbling sometimes.

P9, Group 2

They also stated that gaming could help overcome boredom as an additional rehabilitation tool that a therapist could employ.

The more tools you have to try and get someone motivated or participating in their exercise program, is good. It’s what we are looking for.

P10, Group 2

Another said,

I find there are only so many ways I can make grasp/release entertaining, right? Like blocks, eventually it’s just grasp and release for a really long time.

P9, Group 2

Clients’ experience and familiarity with social media and gaming were thought to influence their motivation to use these technologies for therapy. "I think if it’s something that they’re (the clients) using already that it would be a great motivating tool, but if it’s something that you’re trying to get them into, as well as motivate them to do on top of their exercises, I don’t think it would be as effective." [P7, Group 2].

#### Desirable Features of Gaming Systems for Rehabilitation

A summary of features that the therapists were seeking in gaming systems are described in Textbox 3. Therapists identified the need for future systems to be able to record and report empirical data of clients’ progress in a meaningful way. One feature that was repeatedly mentioned was being able to track a client’s progress in a meaningful way. "Maybe they can automate, so as soon as you get off it sends a report." [P7, Group 2] "It’s empirical data, basically, without you sitting there and counting." [P8, Group 2]

Therapists were seeking gaming systems that offered a variety of games to appeal to different ages, genders, interests, and physical and cognitive abilities, and they desired gaming that incorporated a variety of movements. For example, games that promoted bimanual activities were considered useful. "Yeah, I like the idea of being able to stabilize with one hand and then do another activity because it’s very functional to our daily life." [P4, Group 1] The ability for the system to support grading of task difficulty and allow for success was felt to be important.

there needs to be a level of difficulty that you can make gains but there also has to be success. If you’re just failing all of the time, you give up, and it’s not fun

P3, Group 1

Whether it’s a game for lower functioning individuals…we’re working on just a basic exercise, there need to be maybe six different options within that…

P8, Group 2

Therapists also reported that sensory feedback would be a useful feature to include in a gaming system. "If there would be a vibration or different textures, that would create increased awareness of the affected limbs." [P4, Group 1] There was consensus that positive feedback is an important feature of gaming systems*.*

The positive feedback I think is an important piece because you see they’re working really hard and they’ve had a lot of things happen…there are a lot of barriers for them, so to get some negative feedback from a video game is not a necessary thing for our clients right now. It really needs to be positive.

P5, Group 2

Finally, therapists wanted a system that is simple to set up for themselves and for their clients and appears typical, rather than specific to the rehabilitation setting. "Nothing too complicated, but very simple to use, very simple instructions." [P8, Group 2]. "If you could make it as mainstream as possible." [P2, Group 1] Another therapist noted,

Frustration is often a really big part of their lives, so if the interface and tools are seamless, I think it will be great. But if those are barriers, that can increase the frustration that they already experience regularly.

P4, Group 1

**Desirable features of gaming systems**

Desirable features

- Socio-emotional
  - Ability to play with peers without disabilities
    - “I think this plays in with the social media, but I think peers and having your peers involved is huge.” [P2]
  - Feedback & encouragement
    - “The positive feedback I think is an important piece because you see they’re working really hard and they’ve had a lot of things happen…there are a lot of barriers for them, so to get some negative feedback from a video game is not a necessary thing for our clients right now. It really needs to be positive.” [P5]
  - Variety of games that are developmentally appropriate
    - “…have different sizes of pictures and fonts to make it adaptable.” [P3] ”have like, one-liner instructions rather than long instructions so that it’s very specific.” [P7]
  - Games that appeal to different ages, genders, interests
    - “You need to have a variety of games to match people’s ages and leisure interests. So, offering golf for an older individual and maybe a little bubble flower game for a younger child.” [P5]
- Rehabilitation
  - Capacity to reinforce therapeutic goals
    - “There needs to be a level of difficulty that you can make gains but there also has to be success. If you’re just failing all of the time, you give up, and it’s not fun.” [P3]
  - Capacity to grade movements
    - “Whether it’s a game for lower functioning individuals…we’re working on just a basic exercise, there need to be maybe six different options within that…” [P8]
  - Capacity to target bimanual movements
    - “Yeah, I like the idea of being able to stabilize with one hand and then do another activity because it’s very functional to our daily life.” [P4]
  - System that can overcome compensatory movements
    - “Using sensors or vibration or something to help give feedback when a client is compensating would be helpful.” [P5]
  - Meaningful assessment to track progress
    - “Maybe they can automate, so as soon as you get off it sends a report.”[P7] “It’s empirical data, basically, without you sitting there and counting.” [P8]
- Usability
  - Easy to set up and learn to play
    - “So, just having something that’s very easy to set up. As a therapist, I don’t want to spend more than 10 minutes of my one hour with the client teaching them how to play the game before they even get to play.” [P6]
  - Simple, light, attractive
    - “Nothing too complicated, but very simple to use, very simple instructions.” [P1]
    - “Not too heavy.” [P4]
  - Sensory feedback (vibration, texture)
    - “If there would be a vibration or different textures that would create increased awareness of the affected limbs.” [P4]
  - Mainstream games
    - “If you could make it as mainstream as possible.” [P2]

## Discussion

### Principal Findings

#### Overview

These data highlight the importance of understanding therapists’ perceptions of gaming and social media use by therapy clients. Therapists are key stakeholders who determine the appropriateness of interventions, designing and implementing rehabilitation programs by combining their clinical reasoning with the needs and preferences of their clients. Through this process, they tailor interventions to meet the unique needs of each client, modify the challenge and difficulty as the client’s abilities change, and respond flexibly to the client’s learning and performance needs [2]. Their acceptance or resistance to gaming and social media for rehabilitation can potentially influence the type of care their clients receive and the outcomes from their rehabilitation. These findings indicate the complexities of implementing new technologies in therapy. Despite reporting several challenges with social media and gaming adoption, therapists were open to helping shape the development of interactive gaming systems.

**Figure 1:**
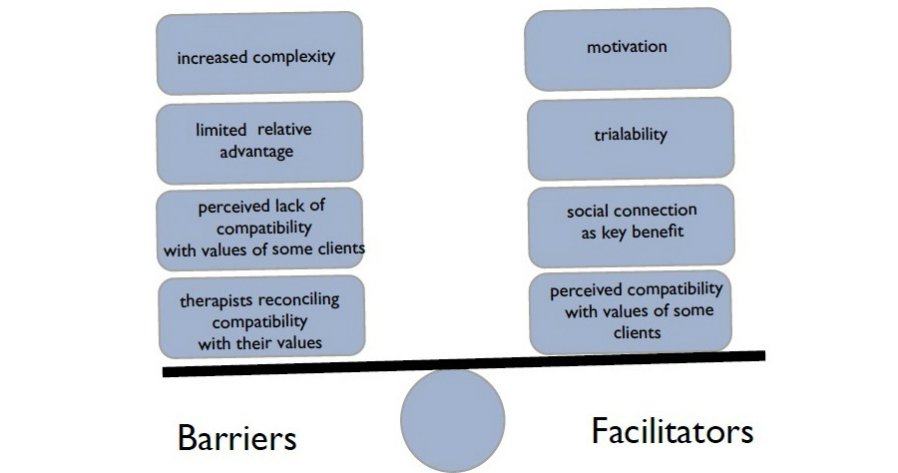
This figure presents a diagram applying diffusion of innovation theory to the focus group themes related to therapists’ adoption of social media and gaming (the innovation) in clinical practice.

The diffusion of innovations model describes five characteristics that most influence the rate of adoption of an innovation [21,25]. The characteristics are (1) relative advantage: the degree to which an innovation is perceived as better than the ideas before it, (2) compatibility: the degree to which an innovation is perceived as being consistent with existing values, experiences, and current needs, (3) complexity: the perception of an innovation as difficult to understand and use, (4) trialability: the degree to which an innovation can be modified and experimented with, and (5) observability: the degree to which the results of the innovation are observable. Figure 1 presents a diagram applying the diffusion of innovation theory to the focus group themes related to therapists’ adoption of social media and gaming (the innovation) in clinical practice.

#### Complexity

Findings from this study suggest that the therapists perceived video gaming and social media systems as complex for their clients and themselves. Complexity barriers included the complexity surrounding client privacy and security issues, maintaining patient confidentiality, and particularly for children, balancing parental needs and comfort levels related to game selection and social media platforms with a child’s sense of autonomy. Importantly, therapists also reported that they perceived that commercial gaming systems presently lack accessibility features to meet the complex and variable needs of clients with hemiplegia.

#### Relative Advantage

Therapists in the focus groups weighed the relative advantage of video gaming and social media use to enhance adherence to exercise programs against traditional therapy programing. For example, therapists in these focus groups perceived that the majority of their adult patients engage in limited use of social media and online gaming; instead, most are using email in their daily lives. Moreover, therapists perceived that children under 13 years old do not use social media, and that privacy issues act as a barrier to its use. Some clinicians considered how to balance therapeutic gaming with other forms of therapy, as they thought that movements produced while gaming are not as effective at improving functional abilities as clients engaging in physical activities outdoors.

All clinicians acknowledged social connectivity as an important potential advantage of social media use, particularly when combined with gaming. They felt that gaming and social media allowed clients to engage with peers in an environment where their impairments are not obvious to others. Some therapists considered social interaction with gaming as a potentially motivating feature that could promote practice and adherence for upper limb therapy.

When conceptualizing video gaming as a therapeutic tool, these findings indicate the need for the benefits to both the clients and the therapists to be clear. Potential gaming and social media benefits to clients can include enhanced socializing with peers, real time feedback, and increased intrinsic motivation [9]. Therapists can also benefit from automated practice of repetitive movements, programmable levels of assistive or resistive training to meet a variety of patient needs, and objective evaluation of the clients motor abilities (including speed, strength, and range of motion) [15]. Although the therapists in this study acknowledged some of these benefits (ie, social connection and intrinsic motivation), other potential benefits of these tools were not identified; rather, therapists reported these characteristics as features they would like to see in future systems. These findings indicate the need to increase therapists’ awareness of current video gaming and social media technologies, and the evidence the feasibility and effectiveness of these specific applications, so that they can make well-informed decisions when considering whether or not to adopt different technologies with their clients. Nonetheless, it important to recognize that the body of evidence in this area does not clearly identify the superiority of video gaming over conventional therapy [26]. A recent systematic review found video gaming therapies to be associated with small but tangible benefits compared to conventional control therapies in adults post stroke [9]. However, this review found studies to date to be generally weak in quality, with the direction and magnitude of these effects dependent on the etiology of the impairment, and functions targeted by therapies (eg, balance versus upper extremity abilities) [26].

#### Compatibility

Therapists appeared to be questioning the compatibility of social media and gaming with the values, past experiences, and needs of their clients and themselves. While recognizing that some clients value specific types of technology, such as iPads, personal computers, and the Wii, therapists perceived that others engage little in technology use. They reported that technology as part of therapy can be a great motivational tool if clients and families already use it in their daily lives.

Therapists identified their role in selecting and adapting games and activities as key to meeting the unique physical, cognitive and socio-emotional needs of each client and supporting the rehabilitation process. They described the inherent clinical reasoning involved in tailoring activities to meet the needs and interests of each client (eg, moving cones), and that they were reconciling how these values fit within delivering interventions using gaming systems. Participants appeared to have concerns about the potential for videogames being used in place of a therapist, rather than as a tool during therapy sessions.

#### Trialability and Observability

The therapists in our study did not perceive that current gaming systems allow them to easily observe and track client progress during gaming-based interventions and were seeking systems that allow them to assess changes in their clients’ abilities. In addition, the therapists seemed to have limited awareness of how social media and gaming can be used in rehabilitation. Some therapists questioned whether gaming therapy could translate to observable functional improvements in a client’s daily life.

#### Implications

This study explored therapists’ perceptions of social media and video game technology in the daily life and rehabilitation of their clients. This study is part of a larger programme of research, Functional Engagement in Assisted Therapy through Exercise Robotics: Intrinsic Motivation Factors (FEATHERS), that is using an integrated knowledge translation approach to develop and to implement an interactive gaming system for upper extremity rehabilitation of hemiplegia in children with CP and in adults secondary to stroke [27]. Thus, these data have important implication for our group (and others) in the development of video games and social media as an assistive technology. For example, a strong potential benefit of games and social media in rehabilitation is the possibility for increased socialization and information transfer between clients and therapists, which is a major component of the FEATHERs project. Clients will have the choice to share their scores and progress with their peers in their own social networks.

Clearly, concerns related to privacy when using social media were raised and need to be addressed when using these systems. Institutional policies that optimize health care delivery for patients are needed to provide guidance in this area [19]. Therapists also expressed interest in the capacity of gaming systems to track client performance as well as ways to detect compensatory and/or cheating motions during therapy. The findings from this study and others [28,29] suggest the benefits of designing interactive gaming systems using a collaborative team-based approach from the outset and consisting of experts that intersect a number of disciplines, including engineers, software and gaming developers, occupational and physical therapists, other rehabilitation professionals, and patients can lead to the development of well designed systems that meet the needs of this client population. While capacity to build features on to motion controlled gaming systems after the fact (eg, add-ons to the Kinect, PS Move, or Wii) is available, clearly identifying this functionality as desirable might allow therapists and other clinicians to start a dialogue with designers to build the functionality into the system (rather than to add on to the system ad hoc).

Understanding and addressing what therapists and clients perceive as barriers to therapy increases the likelihood of adopting the technology and increases the likelihood of continued use. Strategies for promoting clinicians’ adoption of technologies to clinical practice include maximizing desirable features and minimizing barriers to use in the design phase, continuing education and professional development once the technology is released, and mentorship from a clinical opinion leader [30]. In addition, straightforward and easy-to-follow research syntheses or clinical synopses can be developed [31]. Finally, strategies to monitor clinicians’ use of gaming technology are necessary to determine how and the extent to which knowledge has diffused amongst clinicians, and can be used to determine whether or not further knowledge translation is required [32]. Overall, provider acceptance may be more favourable when a balanced use of varied strategies that target work processes, individual knowledge and skills, and formal roles and responsibilities are provided to support implementation [33].

### Limitations

The transferability of these findings is limited by the relatively small sample of participants drawn from rehabilitation settings in BC, Canada. As participants generally worked in urban centres, viewpoints of therapists working in remote or rural areas of the province were not captured. Furthermore, the transferability of findings to individual therapists over 49 years may also be limited by the presence of a single therapist in our sample representing this age range. Nevertheless, recurrent concepts, ideas and themes were found in our groups, indicating that data saturation was achieved with the sample included in our study. Future qualitative studies with a larger sample of therapists representing more diverse age ranges and geographical locations can build upon these initial findings. A strength of this study was that participants included both occupational and physical therapists providing therapy to individuals with hemiplegia across different age groups and in diverse contexts, ranging from hospital, clinic, community and school settings. This study has provided some preliminary information regarding the experiences of clinicians providing rehabilitation to adults and/or children with hemiplegia, demonstrating common themes amongst both client groups. Future qualitative studies can build upon these findings to explore the experiences of therapists working with each client group independently to identify if further considerations for each group are needed.

### Conclusions

Promoting access to therapy and adherence to therapeutic exercises at a sufficient intensity to induce neuroplastic changes is a current challenge for health care providers [34]. Novel interventions and approaches to rehabilitation delivery are needed to achieve these dosages and the inherent reward systems within mainstream gaming and opportunities for social connection with social media render these technologies as potentially valuable tools. Preliminary, compelling evidence exists to suggest positive effects of video gaming on function compared to conventional therapy control groups [3,35-37]. Indeed, a number of studies have explored the application of video gaming technology to promote rehabilitation outcomes [4,9,28,35,38]. Early studies on commercial gaming are suggesting benefits, but more, larger randomized clinical trials RCTs are needed before the clinical efficacy of commercial gaming interventions is understood.

When considering the adoption of innovations, the diffusion of innovations theory highlights the importance of features, such as the complexity, relative advantage, compatibility, trialability and observability of the innovation. The FEATHERS project is using an integrated knowledge translation approach to develop a novel social media and gaming platform for upper limb rehabilitation. Through an iterative and collaborative design process, this qualitative study identifies therapists' concerns, allowing us to address perceived barriers within the system design. Furthermore, the implementation of ongoing knowledge translation strategies can optimize and support the adoption of this technology into clinical practice by more readily meeting the needs of clinicians.

## Abbreviations

CP: cerebal palsy
FEATHERS: Functional Engagement in Assisted Therapy Through Exercise Robotics
